# Stabilization of a Cart Inverted Pendulum: Improving the Intermittent Feedback Strategy to Match the Limits of Human Performance

**DOI:** 10.3389/fncom.2019.00016

**Published:** 2019-04-05

**Authors:** Pietro Morasso, Taishin Nomura, Yasuyuki Suzuki, Jacopo Zenzeri

**Affiliations:** ^1^Robotics, Brain and Cognitive Sciences Department, Center for Human Technologies, Italian Institute of Technology, Genoa, Italy; ^2^Mechanical Science and Bioengineering Department, Graduate School of Engineering Science, Osaka University, Toyonaka, Japan

**Keywords:** Cart Inverted Pendulum, saddle-like instability, intermittent feedback control, phase reset, internal model simulation

## Abstract

Stabilization of the CIP (Cart Inverted Pendulum) is an analogy to stick balancing on a finger and is an example of unstable tasks that humans face in everyday life. The difficulty of the task grows exponentially with the decrease of the length of the stick and a stick length of 32 cm is considered as a human limit even for well-trained subjects. Moreover, there is a *cybernetic* limit related to the delay of the multimodal sensory feedback (about 230 ms) that supports a feedback stabilization strategy. We previously demonstrated that an intermittent-feedback control paradigm, originally developed for modeling the stabilization of upright standing, can be applied with success also to the CIP system, but with values of the critical parameters far from the limiting ones (stick length 50 cm and feedback delay 100 ms). The intermittent control paradigm is based on the alternation of on-phases, driven by a proportional/derivative delayed feedback controller, and off-phases, where the feedback is switched off and the motion evolves according to the intrinsic dynamics of the CIP. In its standard formulation, the switching mechanism consists of a simple threshold operator: the feedback control is switched off if the current (delayed) state vector is closer to the stable than to the unstable manifold of the off-phase and is switched on in the opposite case. Although this simple formulation is effective for explaining upright standing as well as CIP balancing, it fails in the most challenging configuration of the CIP. In this work we propose a modification of the standard intermittent control policy that focuses on the explicit selection of switching times and is based on the phase reset of the estimated state vector at each switching time and on the simulation of an approximated internal model of CIP dynamics. We demonstrate, by simulating the modified intermittent control policy, that it can match the limits of human performance, while operating near the edge of instability.

## Introduction

The manual stabilization of an inverted pendulum hinged on a cart, allowed to shift in a forward/backward manner (shortly CIP: Cart Inverted Pendulum), is an example of the many unstable tasks that humans must face in everyday life. It is indeed a standardized implementation of the well-known stick balancing task, where human subjects enjoy the challenge of stabilizing a rigid stick on their fingertips in the vertically inverted position. Other challenging tasks that share a similar dynamics, although quite different in many respects, are tightrope walking or walking on stilts. Apparently, a different ballgame is the task of upright standing that any healthy adult is capable to manage in an effortless manner, without considering it “challenging” in any sense. However, although this “trivial” skill shares with the other “challenging” tasks the same inverted pendulum biomechanics, it differs in a specific but relevant aspect related to the control strategy, namely the availability of muscle stiffness (more specifically ankle stiffness) as a stabilizing mechanism, a feature which is not physically possible in stick balancing or walking on stilts.

Although the different balancing paradigms mentioned above involve a number of degrees of freedom it is always possible, at least as a first approximation, to focus on a simplified inverted pendulum paradigm (IP) with a single degree of freedom: the ankle joint, in the case of upright standing, or the virtual joint that characterizes the relative motion of the stick on the finger-tip in the stick balancing task. In the former case the neural controller can combine two stabilizing mechanisms, namely co-activation of ankle muscles in order to modulate ankle stiffness, and active generation of ankle torque, on the basis of a feedback control loop driven by sensory feedback of the body sway. In the case of stick balancing, in contrast, the stiffness of the virtual joint is null by definition and the only available control strategy is feedback based. As a matter of fact, the simplicity and availability of a stiffness mechanism has been suggested by some researcher (Winter et al., 1998), supporting the hypothesis that ankle stiffness strategy is sufficient for the stabilization of upright standing, without any need of an additional control loop that is complicated by the significant delay of sensory feedback. Unfortunately, direct measurements of ankle stiffness (Loram and Lakie, 2002; Casadio et al., 2005) as well as the detailed analysis of spindle feedback (van Soest et al., 2003) ruled out the chances of stabilizing upright stance with a pure stiffness strategy. However, stiffness does contribute to stabilization in such paradigm, relieving delayed feedback control of a significant part of the effort. The remaining part, however, must struggle with the curse of instability due to delayed sensory feedback, on top of the intrinsic instability of the inverted pendulum mechanics, exactly as the apparently different IP paradigms mentioned above. The subjective impression of a marked difference, in terms of psychophysical challenge, between upright standing and CIP balancing, may be due to the fact that evolutionary adaptation to bipedal standing in humans had the chance to optimally tune the parameters that allow the apparently seamless integration of “passive” stiffness with “active” delayed feedback control thus making upright standing an apparently trivial action.

Apart from the presence or absence of a stiffness component of the control action, the different IP paradigms differ as regards two other important features: (1) the employed sensory channels (visual, proprioceptive, and vestibular), and (2) the relation between the CoM (the projection of the Center of Mass of the IP on the support base) and the CoP (Center of Pressure, i.e., the centroid of the contact forces exchanged between the IP and the support base). In all cases, the horizontal acceleration of the CoM, with reference to an unstable equilibrium position typical of any IP system, is approximately proportional to the difference between the position of the CoM and the position of the CoP; moreover, the two variables (CoM and CoP position) can switch their role in the control framework, as controlled variable vs. control variable, while maintaining the goal of the control action, namely to avoid falling, which means to keep the CoM position within a limited interval around the equilibrium position.

In standard bipedal upright standing, the CoP is the control variable and its motion is proportional to the variation of the ankle torque related to the activation of the ankle muscles. In stilt standing, which has been studied mainly as regards energetics (Vaida et al., 1981) the position of the CoP is constrained by the environment and cannot be controlled. The same situation characterizes as well-upright standing in reduced/constrained support conditions, such as standing on a narrow bar or on a tight rope: in such case oscillations in the medio-lateral direction are compensated by spreading the control action to a number of joints of the lower and higher limbs in order to restrain as much as possible the overall sway of the CoM around the fixed CoP. Moreover, the period of such oscillations can be lengthened, thus simplifying the control action, by grasping a long balancing bar. In the CIP or the stick balancing task the relative position of the stick CoM with respect to the CoP is the controlled variable: vestibular information does not help in this case whereas vision becomes dominant. In any case, the feedback component of the stabilization process relies on sensory feedback information about the state of the controlled object and the neural controller must overcome multiple sources of instability, in addition to the gravitational toppling action, namely feedback time delays, sensory and motor noise (Milton et al., 2008).

There is ample evidence suggesting the discontinuous nature of the feedback control action, irrespective of the different experimental conditions and different body segments. Consider, for example, the analysis of posturographic patterns (Collins and De Luca, 1993; Morasso and Schieppati, 1999; Morasso and Sanguineti, 2002), EMG signals (Gatev et al., 1999; Loram and Lakie, 2002; Nomura et al., 2013), and the non-uniform character of sway path (Jacono et al., 2004). Several types of neural control have been proposed in recent years: time-delayed feedback with multiplicative noise (Cabrera and Milton, 2002), model predictive controllers with a sensory uncertainty (Mehta and Schaal, 2002; Gawthrop et al., 2011; Loram et al., 2011, 2016; Insperger and Milton, 2014), time-delayed proportional-derivative-acceleration feedback control (Insperger et al., 2012).

Another promising alternative, that was investigated in previous studies specifically for upright standing, is the intermittent time-delayed feedback control policy (referred to as the intermittent-feedback controller or the intermittent-feedback-control strategy in this article), whereby the human body is modeled as a single or a double inverted pendulum (Bottaro et al., 2005, 2008; Asai et al., 2009, 2013; Suzuki et al., 2012). The power of this strategy stems from its ability to take advantage of an “affordance” of the intrinsic dynamics of an inverted pendulum, namely the fact that the upright equilibrium posture with no active feedback is characterized by a saddle-type instability accompanied by a hyperbolic vector field with stable and unstable manifolds in its phase space: when the driving action is switched off, the state vector is attracted to the equilibrium configuration, if the vector is closer to the stable than to the unstable manifold, whereas it is repulsed away in the opposite case. This “affordance” suggests to adopt an alternation paradigm between an “off-phase,” in the former case, and an “on-phase,” based on a simple proportional-derivative feedback of the delayed state vector, in the latter case. Surprisingly, the alternation between the off- and on-phases (although both characterized by unstable dynamics) can lead to overall bounded stability in a robust manner (Bottaro et al., 2008; Asai et al., 2009, 2013; Suzuki et al., 2012). In a recent paper (Yoshikawa et al., 2016) showed that this control policy can be applied with success also to the CIP system providing a robust dynamic stabilization of the inverted stick as well. Moreover, that study demonstrated that such control policy, based on the alternation of on-phases and off-phases, can reproduce features of the stick oscillations that are known to characterize the performance of expert CIP users: (1) the temporal fluctuations of the velocity increments of the stick, which are not Gaussian but exhibit a truncated Lévy distribution (Cabrera and Milton, 2004; Cluff and Balasubramaniam, 2009); (2) the corrective fingertip movements, which alternate between phases with extremely low movement amplitudes and those with high movement amplitudes, according to a power-law distributions of the inter-corrective movement intervals (Cabrera and Milton, 2002).

It is important to note that for the intermittent control policy the feedback control action operating in the on-phase is not intended to push the state toward the ideal equilibrium position, i.e., the origin of the phase space, but to drive the orbit as close as possible to the stable manifold in order to turn off the control action when and if such condition is reached, in such a way to exploit the “affordance” provided by the intrinsic dynamics of the system during the subsequent off-phase. Since this strategy can stabilize upright standing even if the dynamics of the on-phase is unstable when applied continuously, it greatly expands the size of the stability area in the space of control parameters, in comparison with a conventional continuous control paradigm. However the application of this control policy to the CIP task (Yoshikawa et al., 2016) can be successful, in its standard formulation, only if the task is not too challenging: a stick length longer than 50 cm and a feedback delay shorter than 100 ms. In contrast, expert CIP users can perform well also in much more challenging situations, with a pendulum length as short as 32 cm and an overall sensory delay as long as 230 ms (Milton et al., 2016). Should we conclude that the intermittent control policy is not appropriate to reach the human performance limits but is only adequate for less challenging unstable tasks? The main goal of this paper was indeed to falsify this hypothesis, by outlining a plausible extension of the standard intermittent control policy of the CIP task while maintaining the simplicity of the approach. In order to achieve that goal we will first analyze the reasons of the inability of the standard intermittent control policy to match the human limits and will focus, in particular, on the switching rule that supervises the alternation paradigm: in the standard version of the intermittent controller it is a simple threshold mechanism in the state space of the stick, based on delayed sensory information, and the design/learning problem is reduced to the identification of an optimal tuning of the proportional-derivative control parameters that could limit the oscillations around a limit-cycle. If the CIP task is not too challenging, it is indeed possible to identify a region in parameter space that supports bounded stability and thus allows optimal parameter tuning. However, with an increase of the task difficulty the size of that region decreases and ultimately vanishes when approaching the human performance limits. In other words, the problem is that the standard intermittent strategy is functional if the task is not too unstable and ultimately it fails when the delay of the sensory feedback is significantly larger than the intrinsic falling time constant of the inverted pendulum. An additional reason of failure, in a challenging configuration of the task, is the interaction between cart dynamics and stick dynamics during the on-phase: this interaction, together with the short time constant due to a short stick length, contributes to determine the inappropriate termination of the on-phase by the standard switching mechanism and thus the initiation of the off-phase with a state vector of the stick that is far away from the stable manifold and thus is not appropriate for taking advantage of the affordance provided by the saddle dynamics of the inverted stick. The alternative that is proposed in this study is indeed to substitute the statically tuned threshold mechanism of the standard intermittent controller with a dynamic mechanism that focuses directly on the sequence of switching times, by phase-resetting the estimated state vector at each switching time, using a short-term sensorimotor memory for compensating the intrinsic feedback delay, and running a simplified internal model of the CIP dynamics for terminating each on-phase with a state vector as close as possible to the stable manifold.

Generally speaking, phase-resetting is a phenomenon of synchronization of self-sustained oscillatory activity that may characterize populations of neurons (Tass, 2007) or macroscopic behaviors driven by Central Pattern Generators as in the case of locomotion (Yamasaki et al., 2003). In particular, it is well-known that the rhythmic walking pattern can have adaptive sudden phase shifts in response to external perturbations, as the heel strike event. In the case of the CIP model, the underlying self-sustained oscillatory activity is the alternation of off-phases and on-phases intrinsic in the intermittent control paradigm. Moreover, we suggest that the crucial event that may allow the on-going oscillation to maintain bounded stability is the switch time that marks the termination of the on-phase and the initiation of the off-phase; the idea is to phase shift the estimated value of the state vector of the pendulum at that switch time by tapping the short-term memory of delayed estimates. This phase shift is made possible by a second “affordance” related to the off-phase of the intermittent control strategy, namely the possibility to predict the timing and the geometry of the off-phase trajectory. In conclusion, the new intermittent control policy includes a predictive element, intended to defeat the destabilizing effect of the sensory feedback delay, in contrast with the standard policy that does not use any prediction. However, such prediction is not continuous in time but discontinuous as the underlying control action.

## The Model

**Figure 1:**
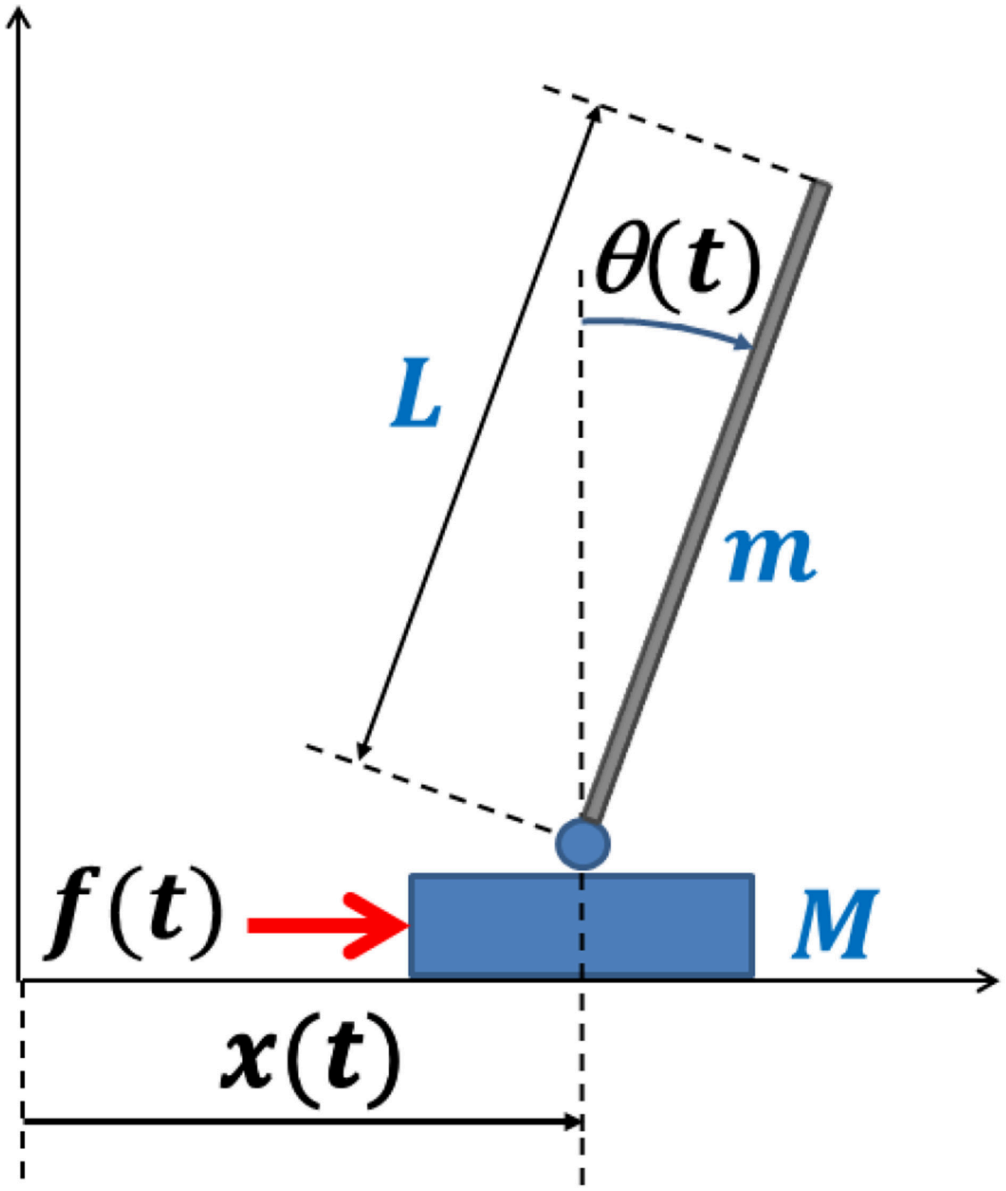
Schematic representation of the CIP model. θ and *x* are the two DoFs; *f* (the control variable) is the force applied by the user to the cart. The stick is a thin uniform rod. The hinge is frictionless as well as the virtual rail that allows the motion of the cart. The *x*-axis is aligned with the antero-posterior direction of the subject's body.

The CIP model is a dynamical system with 2 Degrees of Freedom (DoFs): the cart position *x* and the pendulum angle θ (Figure 1). It is an under-actuated system because the human user has a single control variable, namely the force *f*(*t*) applied to the cart, and thus it is impossible for the user to realize arbitrary trajectories of the two state variables. However, the task of the trained subject is (apparently) simpler: to control *f*(*t*) in order to avoid “fall” over a suitably long interval of time. This means to keep the tilt angle smaller than a given value (in the simulations we used |θ(*t*)| < π/4) while maintaining the position of the cart inside a “reachable interval” (|*x*(*t*)| < Δ*x*) that depends on the fact that the subject is sitting or standing or other physical arrangements.

The CIP system is feedback controlled, i.e., the Central Nervous System generates the output motor variable *f*(*t*) by relying on sensory feedback about the state of the cart $\left[\theta ,\dot{\theta },\;x,ẋ\right]$: this sensory information is multi-modal (vision + proprioception), delayed (delay δ) and noisy. Both elements, namely delay and noise, tend to set limitations to the performance of human subjects, reducing their capability to avoid the fall of the pendulum. In the simulations carried out for this study the feedback delay is set to 230 ms, taking into account the experimental evaluations of Milton et al. (2016). The sensory feedback uncertainties are modeled as an additive Gaussian noise, as in Yoshikawa et al. (2016), which is added to the control force *f*(*t*): *f*_*n*_(*t*) = σ ξ (*t*), where ξ(*t*) represents a Gaussian white noise with zero mean and unit variance and σ is the noise intensity (standard deviation of the noise).

The CIP system parameters are the pendulum length *L*, the pendulum mass *m*, and the cart mass *M*. From the point of view of task difficulty *L* is the critical parameter. As reported by Milton et al. (2016) a length of 32 cm is the limit for human subjects. We chose this value for the simulations. As regards the other two parameters we adopted the same values used by Yoshikawa et al. (2016): *m* = 0.125 kg, *M* = 2 · *m* = 0.25 kg.

The dynamics of the CIP system is governed by the following non-linear dynamic equations (see the Supplementary Material for details):

(1) $$\begin{array}{l} \left[\begin{array}{c} \ddot{\theta }\\ ẍ \end{array}\right]=\left[\begin{array}{cc} A_{11}\left(\theta \right) & A_{12}\left(\theta \right)\\ A_{21}\left(\theta \right) & A_{22}\left(\theta \right) \end{array}\right]\left[\begin{array}{c} \sin\vartheta \\ f \end{array}\right] \end{array}$$

where the matrix elements are functions of the pendulum angular tilt (*g* is the gravity acceleration):

(2) $$\begin{array}{l} \left\{ \begin{array}{l} A_{11}=\frac{1.5}{L(M+m(1-0.75{\text{cos}}^{2}\theta ))}((M+m)g-0.5mL{\dot{\theta }}^{2}\text{cos}\theta )\\ A_{12}=\frac{-1.5\text{cos}\theta }{L(M+m(1-0.75{\text{cos}}^{2}\theta ))}\\ A_{21}=\frac{1}{M+m(1-0.75{\text{cos}}^{2}\theta )}(0.5mL{\dot{\theta }}^{2}-0.75mg\text{cos}\theta )\\ A_{22}=\frac{1}{M+m(1-0.75{\text{cos}}^{2}\theta )} \end{array}\right. \end{array}$$

Although the simulations considered in the results section use the non-linear model above, for stability analysis and for managing the alternation between on- and off-phases a linearized model is used, in the neighborhood of the origin, described by the following equations:

(3) $$\begin{array}{l} \left[\begin{array}{c} \ddot{\theta }\\ ẍ \end{array}\right]=\left[\begin{array}{cc} A_{11} & A_{12}\\ A_{21} & A_{22} \end{array}\right]\left[\begin{array}{c} \vartheta \\ f \end{array}\right] \end{array}$$

with the following constant matrix elements:

(4) $$\begin{array}{l} \left\{ \begin{array}{l} A_{11}=\frac{1.5(M+m)}{\left(M+0.25m\right)}\frac{g}{L}\\ A_{12}=-\frac{1.5}{(M+0.25m)L}\\ A_{21}=-\frac{0.75mg}{M+0.25m}\\ A_{22}=\frac{1}{M+0.25m} \end{array}\right. \end{array}$$

By looking at Equations 1 or 3 it is immediate to observe that, in the absence of control action, the motion of the pendulum is independent of the motion of the cart. Moreover, in the case of the linearized model, such motion is characterized, in the phase plane of the pendulum ($\theta vs\dot{\theta }$), by an instability of the saddle type, with two real eigenvalues of opposite signs ($\lambda =\pm \sqrt{A_{11}}$). The corresponding eigenvectors identify, respectively, a stable manifold ($\dot{\theta }=-\sqrt{A_{11}}\theta$), namely a line whose half-line trajectories converge to the origin, and an unstable manifold ($\dot{\theta }=+\sqrt{A_{11}}\theta$), namely a line whose half-line trajectories diverge from the origin: the unstable manifold spans the first and third quadrants of the phase plane and the unstable manifold spans the second and fourth quadrants.

### The Standard Intermittent Control Policy of the CIP Model Based on Optimal Tuning of the Feedback Control Parameters

The intermittent stabilization strategy was originally developed for modeling the stabilization of upright standing, when representing the standing body as a single DoF inverted pendulum (Bottaro et al., 2005, 2008; Asai et al., 2009, 2013; Suzuki et al., 2012). In that case the control variable is the ankle torque τ, whereas in the CIP system it is the force *f* applied to the cart. In both cases, however, there is an alternation of *on-phases*, where the control action is provided by a simple Proportional/Derivative (*PD*) delayed feedback error mechanism, and *off-phases*, where the control action is switched off. The error signals, for the CIP system, are the differences of the two DoFs (θ, *x*) from the corresponding reference values (θ_*ref*_ = 0; *x*_*ref*_ = 0) and the control action is characterized by two proportional parameters: (*P*_θ_, *P*_*x*_) and two derivative parameters (*D*_ω_, *D*_*v*_). In short, the standard version of the intermittent control policy is summarized by the script of Box 1:

Box 1 **Standard version of the Intermittent control policy**

**On-phase**

**Activating condition:** $\theta \left(t-\delta \right)\left[\dot{\theta }\left(t-\delta \right)-a\;\theta \left(t-\delta \right)\right]<0$

**Control Action:** ${f\left(t\right)=P}_{\theta }\theta \left(t-\delta \right)+D_{\omega }\;\dot{\theta }\left(t-\delta \right)+P_{x}\;x\left(t-\delta \right)+D_{v}\;ẋ\left(t-\delta \right)$

**Off-phase**

**Dis-activating condition:** $\theta \left(t-\delta \right)\;\left[\dot{\theta }\left(t-\delta \right)-a\theta \left(t-\delta \right)\right]\geq 0$

**Control Action:** *f*(*t*) = 0

This control policy should be compared with the corresponding continuous control model characterized by the following equation, active all the time:

(5) $$\begin{array}{l} {f\left(t\right)=\hat{P}}_{\theta }\;\theta \left(t-\delta \right)+{\hat{D}}_{\omega }\;\dot{\theta }\left(t-\delta \right)+{\hat{P}}_{x}\;x\left(t-\delta \right)+{\hat{D}}_{v}\;ẋ\left(t-\delta \right) \end{array}$$

The stability analysis of this control policy, carried out by Yoshikawa et al. (2016), demonstrated that asymptotic stability can be achieved provided that the feedback delay satisfies the following condition:

(6) $$\begin{array}{l} \delta <\sqrt{\frac{L}{g}} \end{array}$$

In particular, for *L* = 32 *cm* we have δ < 180 *ms* and this means that the continuous control policy has no chance of stabilizing the CIP system with such stick length and a feedback delay beyond 200 ms. But also the standard intermittent control policy could fail in such conditions for the reasons that we explain in the following.

In the standard intermittent control policy the switching rule between the two phases is formulated in the phase space of the pendulum ($\theta \;vs\;\dot{\theta }$) and divides the plane into two areas, namely the on-area and the off-area. The off-area includes the second and fourth quadrants plus/minus an angular slice, whose amplitude is a function of the parameter *a*, whereas the on-area includes the first and third quadrants minus/plus the same angular slice. In the following we assume for simplicity that *a* = 0 and thus the angular slice disappears.

During an off-phase, initiated at *t* = *t*_*off*_ either in the second or the fourth quadrant of the phase space, the orbit of the state vector will follow a hyperbolic trajectory that initially approaches the origin arriving at a minimum distance (at *t* = *t*_*c*_) when the trajectory intersects one of the two coordinate axes, thus entering one of the other two quadrants influenced by the unstable manifold: thereafter the trajectory will diverge while approaching the unstable manifold. The initial part of the hyperbolic trajectory (up to *t* = *t*_*c*_) is the “affordance” provided by the intrinsic dynamics of the inverted pendulum: during that time there is no need to force the system with active control because mechanics itself carries out the job of fighting the danger of falling. On the other hand, the switching rule is not applied to the current state vector $\left[\theta \left(t\right),\dot{\theta }\left(t\right)\right]$ but to the corresponding delayed sample $\left[\theta \left(t-\delta \right),\dot{\theta }\left(t-\delta \right)\right]$, thus the off-phase will be terminated not at the time of crossing the border between the stable and unstable area but δ milliseconds later: *t*_*on*_ = *t*_*c*_+δ.

**Figure 2:**
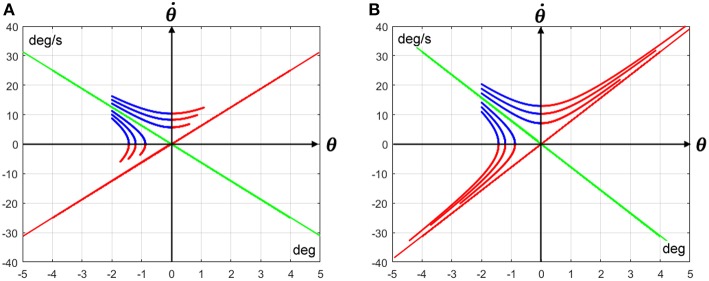
Off-phase trajectories of the CIP model with the same initial angular tilt (−2 deg) but different distance from the stable manifold: the green line (the red line is the corresponding unstable manifold). The blue part of each trajectory runs from the initial position (at *t* = *t*_*off*_) until the intersection with one of the two coordinate axes (at *t* = *t*_*c*_: the duration of such segment is determined by Equation 9); the second part is red-colored and has a fixed duration, equal to the sensory feedback delay δ (*t*_*on*_ = *t*_*c*_+δ). **(A)** Refers to a CIP model with the following parameters: *L* = 50 *cm*; δ = 100 *ms*). **(B)** Refers to a CIP model with much more challenging parameters: *L* = 32 *cm*; δ = 230 *ms*. In both cases the cart mass is 250 g and the stick mass is 125 g.

The problem, as exemplified in Figure 2, is that the timing of the hyperbolic trajectories, as well as the relative position of the state vector at *t*_*on*_ with respect to the position at *t*_*off*_, strongly depend on the initial distance of the state vector from the stable manifold and on the main parameters of the CIP system, namely stick length *L* and feedback delay δ. In Figure 2 all the hyperbolic trajectories initiate with the same angular tilt but with different distances from the stable manifold: the initial blue segment terminates when it intersects one of the two coordinate axes and the following red segment terminates after a fixed time interval equal to the sensory feedback delay δ, namely when the activation condition turns on. Figure 2A refers to the CIP model investigated by Yoshikawa et al. (2016) with the following parameters: *L* = 50 *cm* and δ = 100 *ms*. Figure 2B refers to a much more challenging CIP model, with *L* = 32 *cm* and δ = 230 *ms*. These graphs clarify that for the same initial angular tilt of the pendulum the final position of the state vector will end up further and further away from the origin, as the initial distance from the stable manifold increases, and this potentially diverging pattern emerges clearly in the second configuration of the CIP model that, as observed above, represents the upper limit of human performance.

In the standard intermittent control policy the on-phase is initiated δ seconds after the state vector of the stick has entered the unstable area, at time *t* = *t*_*on*_. Thereafter, the orbit of the state vector will follow an expanding spiral or nodal course, as a function of the *PD* parameters of the stick (*P*_θ_, *D*_ω_), if the *PD* parameters of the cart (*P*_*x*_, *D*_*v*_) are null; moreover, such unstable behavior of the inverted stick is further amplified by including the cart component in the control action. The purpose of this component is indeed to restrain the range of oscillation of the cart to a small feasible value but from the point of view of stick balancing it is an additional source of instability. In any case, the forced orbit of the state vector will ultimately cross a coordinate axis at *t* = *t*_*c*1_, leaving the on-area, and will be terminated, thus initiating the next off-phase, at *t* = *t*_*off*_ = *t*_*c*1_+δ. In short terms, the evolution of the state vector of the stick will be shaped as an alternation of segments of hyperbolic orbits in the off-condition and segments of expanding spiral or nodal orbits in the on-condition with the following timing:

(7) $$\begin{array}{l} t_{off}\to t_{c}\to t_{on}=t_{c}+\delta \to t_{c1}\to t_{off}=t_{c1}+\delta \to \ldots \end{array}$$

The orbits of the off-phases only depend on the CIP parameters (*L, M*, and *m*) whereas the orbits of the on-phases also depend on the control parameters and the motion of the cart, due to the feedback of the control policy. The chance of success of the standard intermittent control policy is determined by the choice of the *PD* parameters and, in particular, by the fact that such tuning may induce a distribution of state vectors at *t* = *t*_*off*_ centered as much as possible on the stable manifold and with a very narrow standard deviation. In order to clarify this point, let us use the following parameter for measuring the distance of the state vector from the stable manifold at *t* = *t*_*off*_:

(8) $$\begin{array}{l} \gamma_{off}=|\frac{\dot{\theta }\left(t_{off}\right)}{\theta \left(t_{off}\right)\sqrt{A_{11}}}| \end{array}$$

γ_*off*_ = 1 means that the state vector is “on” the stable manifold, i.e., the distance is null; γ_*off*_ > 1 means that the state vector is above the stable manifold and γ_*off*_ < 1 that it is below it. The average value of this parameter should be as close as possible to 1, with a suitably small standard deviation. The target of the intermittent control policy indeed is not the equilibrium point, i.e., the origin, but the whole stable manifold at the end of the on-phases. If the *PD* parameters are optimally tuned the value of γ_*off*_ on average will be sufficiently close to 1 to induce hyperbolic segments with contracting properties, i.e., with a distance from the origin at *t* = *t*_*on*_ smaller than the distance at *t* = *t*_*off*_. Such contracting properties of the off-phases may compensate, on average, the expanding properties of the spiral/nodal segments during the on-phases, supporting the emergence of limit-cycle oscillations. As a matter of fact, the study by Yoshikawa et al. (2016) demonstrated that this kind of bounded stability can be achieved with a stick length of 1 m and a sensory delay of 100 ms. On the other hand, this is not possible in the human limit conditions (stick length of 32 cm and sensory delay of 230 ms). In order to better understand the reasons of this failure of the standard intermittent control policy let us focus our attention on the kinematics of the stick during the off-phases. In the Supplementary Material we demonstrate that during the off-phase, initiated at *t* = *t*_*off*_, the time required by the hyperbolic trajectory of the state vector to cross the pertinent coordinate axis, at *t* = *t*_*c*_, is well-approximated by the following equation, which is derived from the linearized CIP model of Equation 3:

(9) $$\begin{array}{l} \Delta t_{cross}=t_{c}-t_{off}=\frac{1}{2\sqrt{A_{11}}}\ln\left(\frac{1+\gamma_{off}}{\left|1-\gamma_{off}\right|}\right) \end{array}$$

The time interval computed by this formula does not depend on the initial tilt angle *per se* but on the “distance” from the stable manifold, measured by the value of γ_*off*_: it strongly increases as the distance of the starting point from the stable manifold decreases, ultimately diverging when it becomes zero. The reason is that, in such case, the starting point is exactly on the stable manifold and the hyperbolic trajectory degenerates to the line of the corresponding manifold; moreover, the crossing points coincides with the origin and is reached asymptotically following an exponential descent.

**Figure 3:**
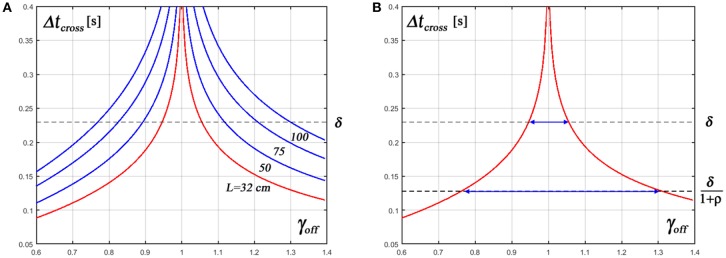
Characteristic timing of the hyperbolic trajectories in the off-phases. Δ*t*_*cross*_ is the time taken by an hyperbolic trajectory to cross the border, between the off-area and the on-area, as a function of the distance of the starting point $\left[\theta_{0},\;{\dot{\theta }}_{0}\right]$ from the stable manifold ($\dot{\theta =-\sqrt{A_{11}}\;\theta }$). Such distance is measured by the parameter $\gamma_{off}=|\frac{{\dot{\theta }}_{off}}{\theta_{off}\sqrt{A_{11}}}|.\;\gamma_{off}=1$ means that the starting point of an off-phase is exactly on top of the manifold and in this case the crossing time diverges, whereas it quickly decreases with the increase of |γ_*off*_ − 1|. **(A)** Displays Δ*t*_*cross*_ as a function of γ_*off*_ for different values of the stick length and feedback delay δ = 230 ms. Since the stability condition of the off-phase for the standard intermittent control policy is Δ*t*_*cross*_ > δ, the graph clearly shows that the interval of values of γ_*off*_ that support such condition strongly decreases with the shortening of the stick length. **(B)** Focuses on the most challenging configuration of the CIP balancing task (*L* = 32 *cm*, δ = 230 *ms*) and compares the range of values of γ_*off*_ that support stability in the standard and in the new intermittent control policy (Δ*t*_*cross*_ > δ vs. Δ*t*_*cross*_ > δ/(1+ρ), respectively). ρ = 0.8 is the “contraction factor”.

The graph of Figure 3A plots the variation of the time to cross described by Equation 9, computed for the most critical value of the sensory delay time (δ = 230 *ms*) and for different values of the stick length. It clearly shows that, with decreasing values of the stick length, the interval of values of γ_*off*_ that are compatible with a contracting pattern of the off-phase strongly decreases. We should consider indeed that the total duration of the hyperbolic trajectory for a given off-phase, with the switching rule of the standard intermittent policy, is as follows:

(10) $$\begin{array}{l} \text{Duration of the off-phase}:\;\Delta t_{cross}+\delta \end{array}$$

Moreover, since the hyperbolic trajectories of the off-phase are approximately symmetric with respect to the intersected coordinated axis, the condition that the off-phase orbit is not expanding (a sufficient condition for stability) is as follows:

(11) $$\begin{array}{l} \Delta t_{cross}>\delta \Rightarrow t_{c}-t_{off}>t_{on}-t_{c} \end{array}$$

The graph of Figure 3A shows that for a stick length of 100 cm the condition above requires that the initial distance of the state vector from the stable manifold |γ_*off*_ − 1| is about ±0.3; for a stick length of 50 cm the distance should be < ±0.1 and for the limit case of the 32 cm stick the distance should be even smaller (±0.05). We also emphasize that, even with an optimal tuning of the feedback parameters, the distance from the stable manifold at the end of the on-phase will be spread in a range strongly growing with the decrease of the stick length, as a consequence of the sensory noise and the disturbing effect of the cart motion. For this reason the simple switching mechanism of the standard intermittent control policy is doomed to fail at some level of difficulty of the task and this may suggest to the trained subject a modification of the intermittent control policy, focusing on the optimal tuning of the switching times rather than the *PD* control parameters.

The Supplementary Material, in addition to Equation 9, provides also the derivation of the following equation, which describes the full course of the stick trajectory in the off-phase, and, in particular, can be used in order to predict the state of the stick at the time of termination, i.e., at *t* = *t*_*on*_:

(12) $$\begin{array}{l} \left\{ \begin{array}{l} \theta (t)=\frac{{\dot{\theta }}_{off}+\theta_{off}\sqrt{A_{11}}}{2\sqrt{A_{11}}}e^{\sqrt{A_{11}}(t-t_{off})}+\frac{-{\dot{\theta }}_{off}+\theta_{off}\sqrt{A_{11}}}{2\sqrt{A_{11}}}e^{-\sqrt{A_{11}}(t-t_{off})}\\ \dot{\theta }(t)=\frac{{\dot{\theta }}_{off}+\theta_{off}\sqrt{A_{11}}}{2}e^{\sqrt{A_{11}}(t-t_{off})}-\frac{-{\dot{\theta }}_{off}+\theta_{off}\sqrt{A_{11}}}{2}e^{-\sqrt{A_{11}}(t-t_{off})} \end{array}\right. \end{array}$$

Moreover, let us consider the disturbing effect of the cart motion on the dynamics of the pendulum, i.e., the cross-coupling between the cart and the pendulum dynamics during the on-phase. Suppose indeed that the *PD* pendulum parameters were optimally tuned, in such a way to drive the pendulum state vector, in the absence of cart control, on top of the stable manifold at *t* = *t*_*off*_, which is the ideal situation for exploiting the stabilizing effects of the off-phase dynamics. However, even in this case, a minimum amount of drive of the cart motion, just sufficient to maintain the cart position in a feasible range, will induce a variability of the initial state vector ($\theta \left(t_{off}\right),\dot{\theta }\left(t_{off}\right)$ that, given the strong non-linearity of Equation 9, will inevitably trigger a transition to instability: the larger the error (i.e., the distance of γ_*off*_ from the target value of 1) the quicker will be the descent of the undriven hyperbolic trajectory with the danger of over-penetrating the on-region and thus enlarging more and more the composite orbit away from equilibrium.

### The New Intermittent Control Policy of the CIP Model Based on On-Line Selection of the Switching Times

In the standard intermittent control strategy, the sequence (*t*_*on*_, *t*_*off*_, *t*_*on*_, *t*_*off*_, …) of switching times for activation/dis-activation of the delayed feedback control is an *indirect effect* of the choice of control parameters and thus there is no guarantee that when active control is switched off the state vector is close enough to the stable manifold, in such a way to produce a sequence of hyperbolic-spiral-hyperbolic-spiral-…. oscillatory segments of the inverted stick approaching a limit-cycle of the unstable equilibrium point. However, if the stick is sufficient long (e.g., 0.5 m) it is possible to identify a range of control parameters that indirectly produce a bounded stability, as demonstrated by Yoshikawa et al. (2016). As a matter of fact, falling is what happens frequently to naïve subjects who typically need a long training exercise for a CIP configuration near the limit conditions defined above. We suggest that this achievement can be obtained by building an internal model of on-line adaptation that complements, for the more challenging configurations of the system, the static parametric optimization of the standard intermittent control policy. The crucial step, in our opinion, is to focus the attention of such “cybernetic supervisor” on the *explicit selection* of switching times in relation with the corresponding sequence of on-phase and off-phase trajectories.

There are indeed two crucial events in the sequence that need to be optimized in order to avoid the spiraling away of the CIP oscillatory patterns:

- The termination of the hyperbolic trajectories of the off-phases, i.e., the *explicit selection of t*_*on*_, in order to avoid “over-penetration” of the state vector in the potentially dangerous area;
- The termination of the unstable spiral-like trajectories of the on-phases, i.e., the *explicit selection of t*_*off*_, in order to switch off the control action when the state vector is as close as possible to the stable manifold. The critical importance of minimizing the distance of the state vector from the stable manifold at *t*_*off*_ is that the overall speed of the hyperbolic trajectories in the off-phase strongly increases with such distance (as shown in Figure 3) and thus the rationale of the intermittent control policy, namely exploiting the self-stabilizing affordance of the off-phases, is ruled out unless the speed of the hyperbolic trajectories is appropriately limited.

In summary, the script of the standard version of the intermittent control policy is substituted by the following one (Box 2), taking into account that the explicit selection mechanisms of *t*_*on*_ and *t*_*off*_, respectively, that will be examined in the following sections:

Box 2 **New version of the Intermittent control policy**

**On-phase** *t*_*on*_ < *t* < *t*_*off*_

**Termination:** explicit selection of *t*_*off*_ via internal simulation of time-reset internal model

**Control Action:** ${f\left(t\right)=P}_{\theta }\theta \left(t-\delta \right)+D_{\omega }\dot{\theta }\left(t-\delta \right)+P_{x}x\left(t-\delta \right)+D_{v}ẋ\left(t-\delta \right)$

**Off-phase** *t*_*off*_ < *t* < *t*_*on*_

**Termination:** explicit selection of *t*_*on*_ via evaluation of γ_*off*_ and Δ*t*_*cross*_

**Control Action:** *f*(*t*) = 0

#### Terminating the Hyperbolic Trajectory of the Off-Phase by an Explicit Selection of **t**_**on**_

At the termination of the on-phase, i.e., when the time stamp *t* = *t*_*off*_ is instantiated, the hyperbolic trajectory is started but the actual position in the phase space of the state vector of the stick $\sigma_{off}=\left[\theta \left(t_{off}\right),\dot{\theta }\left(t_{off}\right)\right]$ is unknown because the control system has direct access only to the delayed state which may be markedly different from the real one. The knowledge of σ_*off*_ is not relevant for the neural control of the hyperbolic off-phase trajectory, which is fully determined by the physics of the CIP system, but it is crucial for the explicit selection of *t*_*on*_ and for the prediction of the corresponding initial state of the on-phase:

$$\begin{array}{l} \sigma_{on}=\left[\theta \left(t_{on}\right),\dot{\theta }\left(t_{on}\right)\right]. \end{array}$$

A key idea of the new intermittent control policy is that σ_*off*_ can be recovered in a natural way not at *t* = *t*_*off*_ but at *t* = *t*_*off*_+δ by assuming that the neural controller has access to the short-term sensory-motor memory of the trajectory of the stick: the initially unknown position will indeed become available δ seconds later by directly tapping the delayed sensorimotor information:

$$\begin{array}{l} \theta_{off}=\theta \left[\left(t_{off}-\delta \right)+\delta \right];\;{\dot{\theta }}_{off}=\dot{\theta }\left[\left(t_{off}-\delta \right)+\delta \right]. \end{array}$$

With this geometric information it is then possible to estimate Δ*t*_*cross*_, that characterizes the descending part of the hyperbolic trajectory, up to Δ*t* = *t*_*c*_, by using Equations 8 and 9, without any interference of the concurrent cart motion. Moreover, with such timing information it is possible to choose the appropriate termination time of the off-phase by setting up a timer at a future time instant *t* = *t*_*on*_, thus concluding the explicit selection of the off-phase temporal sequence: *t*_*off*_ → *t*_*c*_ → *t*_*on*_. More specifically, the terminal time should be selected in such a way to induce a contracting effect of the off-phase trajectory, i.e., |σ_*on*_| < |σ_*off*_| and this effect can be easily achieved with the following choice:

(13) $$\begin{array}{l} t_{on}=t_{c}+\Delta t_{cross}\cdot \rho \end{array}$$

where ρ is the “contraction factor” (in the simulations we used a value of 0.8 but the specific value is not critical for stability, provided that it is < 1). In summary, the computational process for exploiting in the best way the self-balancing properties of the off-phase can be described by the following script (Box 3):

Box 3 **Explicit selection of *t*_*on*_ in the new version of the Intermittent control policy**

- After turning-off the active control at *t* = *t*_*off*_, wait a time interval δ and tap $\sigma_{off}=\left[\theta \left(t_{off}\right),\dot{\theta }\left(t_{off}\right)\right]$ out of the short-term sensorimotor memory at *t* = *t*_*off*_ + δ;
- From σ_*off*_ evaluate Δ*t*_*cross*_ by using Equations 8 and 9;
- Setup up a timer at a future time instant *t* = *t*_*on*_, selected according to Equation 13;
- Anticipate the predicted state vector at *t* = *t*_*on*_, $\hat{\sigma }\left(t_{on}\right)$, by using Equations 12.

It is important to highlight that the first step of the script plays the role of *phase*-*resetting* the time course of the measured stick angular oscillation, compensating at least locally the intrinsic feedback delay. However, the contracting pattern of the off-phase trajectory, namely that |σ_*on*_| < |σ_*off*_|, can occur if and only if the following condition is met:

(14) $$\begin{array}{l} t_{on}-t_{off}>\delta \end{array}$$

This is also equivalent to the following condition on the time to cross of the hyperbolic trajectories and, ultimately, on the corresponding initial distance of the state vector from the stable manifold:

(15) $$\begin{array}{l} \Delta t_{cross}>\frac{\delta }{1+\rho } \end{array}$$

Such stability condition of the new intermittent control policy should be compared with the corresponding condition of the standard policy:

(16) $$\begin{array}{l} \Delta t_{cross}>\delta \end{array}$$

We may conclude (see also Figure 3B) that the new intermittent control policy is much more robust than the standard policy as regards the regulation of the off-phase in order to guarantee that |σ_*on*_| < |σ_*off*_| because it can tolerate a much larger range of values γ_*off*_, i.e., a greater inaccuracy in the termination of the on-phase in terms of distance of σ_*off*_ from the stable manifold.

#### Terminating the On-Phase by Running an Internal Model of the Forced Dynamics for the Explicit Selection of **t**_**off**_

After activation of the feedback control signal at *t* = *t*_*on*_, the orbit of the pendulum state vector will spiral away from the unstable manifold intersecting first the *x*-axis and then the stable manifold. The latter event is the crucial piece of information for terminating the activation phase in the optimal way, i.e., for allowing to exploit in the best possible way the stabilization affordance of saddle dynamics ($\dot{\theta }=-\sqrt{A_{11}}\;\theta$). The problem is that detecting this event is far from trivial: while the evolution of the hyperbolic trajectory in the off-phase is fully predictable and can be computed by taking advantage of an explicit equation, no such formula is available in the on-phase mainly for the disturbing effect of the cart dynamics on the dynamics of the pendulum. On the other hand, attempting to detect the intersection directly by means of the delayed sensory information is likely to be very imprecise for the high falling speed of the 32 cm stick. The proposed solution is to run a simulation of a simplified internal model of the forced CIP dynamics, for *t* > *t*_*on*_, using Equations 3: the simulation is initialized with the predicted value of the pendulum state vector at *t* = *t*_*on*_, i.e., $\hat{\sigma }\left(t_{on}\right)$, made available by the phase reset of the stick oscillation pattern explained in the previous section. Such simulation will generate an approximated but un-delayed version $\hat{\theta }\left(t\right)$ of the real trajectory of the stick that can be used for terminating the off-phase. Summing up, the explicit selection of *t*_*off*_ in the new version of the intermittent control policy is characterized by the following script (Box 4):

Box 4 **Explicit selection of *t*_*off*_ in the new version of the Intermittent control policy**

- At *t* = *t*_*on*_ initialize the internal simulation model with $\hat{\sigma }\left(t_{on}\right)$;
- For *t* > *t*_*on*_ carry out the internal simulation by integrating the linearized dynamical model of Equation 3, producing an un-delayed but approximated trajectory of the stick $\hat{\theta }=\hat{\theta }\left(t\right)$;
- Stop the simulation at a time instant *t*_*s*_ when $\hat{\theta }\left(t_{s}\right)$ crosses the stable manifold
- Select that instant as *t*_*off*_.

#### Simulation of the New Intermittent Control Policy

The simulations were carried out with Matlab (MathWorks), using the forward Euler method with a time step of 1 ms. The control force *f*(*t*) includes an additive noise term: a Gaussian white noise with zero mean and standard deviation equal to 0.015 N. Such noise intensity is similar to the average noise intensity used by Yoshikawa et al. (2016) for the standard intermittent control model.

Another source of uncertainty is related to the estimate of the slope of the stable manifold, which is required by the new intermittent control policy for terminating the on-phase. We modeled such uncertainty with a zero mean Gaussian white noise in order to induce a 20–30% variability of the slope value. In this manner the intersection of the internal model simulation with the stable manifold will be randomized, triggering off-phase trajectories with different values of γ_*off*_. This uncertainty incorporates also the influence of the inaccuracy of the simplified internal model of CIP dynamics because both sources of uncertainty (the one related to the slope and the other to the internal model) only matter as long as they co-influence the misselection of the switching time from the on-phase to the off-phase.

As regards the *PD* parameters of the stick (*P*_θ_, *D*_ω_) we identified rough initial estimates by considering the linearized model equations of Equation 3, while ignoring the influence of the cart on the stick dynamics:

(17) $$\begin{array}{l} \ddot{\theta }=A_{11}\theta +A_{12}\;f\approx A_{11}\theta +A_{12}\left(P_{\theta }\;\theta \left(t-\delta \right)+D_{\omega }\dot{\theta }\left(t-\delta \right)\right) \end{array}$$

The delayed state vector was approximated with the first order Taylor's expansion^1^:

(18) $$\begin{array}{l} \left\{ \begin{array}{l} \theta (t-\delta )\sim \theta (t)-\dot{\theta }(t)\delta \\ \dot{\theta }(t-\delta )\sim \dot{\theta }(t)-\ddot{\theta }(t)\delta \end{array}\right. \end{array}$$

This provides the following approximated, linearized equation of the on-phase

(19) $$\begin{array}{l} \ddot{\theta }\left(1+A_{12}D_{\omega }\delta \right)+\dot{\theta }\left(A_{12}P_{\theta }\;\delta -A_{12}D_{\omega }\right)+\left(-A_{11}-A_{12}P_{\theta }\right)=0 \end{array}$$

The requirements for asymptotic stability of such model are then as follows:

(20) $$\begin{array}{l} \left\{ \begin{array}{l} D_{\omega }<\frac{(M+0.25m)L}{1.5\delta }\\ D_{\omega }>P_{\theta }\delta \\ P_{\theta }>(M+m)g \end{array}\right. \end{array}$$

Moreover, we can obtain an estimate of the limit critical value of the time delay for achieving such asymptotic stability:

(21) $$\begin{array}{l} \delta_{crit}=\sqrt{\frac{\left(M+0.25m\right)L}{1.5\left(M+m\right)g}} \end{array}$$

With the model parameters used in this study the critical value of the time delay is 128 ms and thus, with the considered delay of 230 ms, it will be impossible to satisfy all the three conditions above at the same time, in particular the first and the second one. However, by choosing the two *PD* parameters in such a way to satisfy the first and the third conditions we will be confident that the trajectories of the on-phase will be characterized either by an unstable node or spiral:

(22) $$\begin{array}{l} \left\{ \begin{array}{l} D_{\omega }<0.261\;Ns/rad\\ P_{\theta }>3.678\;N/rad \end{array}\right. \end{array}$$

In particular, in the simulations we used the value *D*_ω_ = 0.1686 *Ns*/*rad* for the former parameter and we varied, the latter, in the following range: *P*_θ_ = 4 ↔ 20 *N*/*rad*.

The choice for the *PD* parameters of the cart (*P*_*x*_, *D*_*v*_) was guided by two conflicting requirements:

- To choose values as small as possible in order to minimize the disturbing effects on the stick stabilization due to the dynamics of the cart;
- To limit the range of the cart motion to a physiological level.

In particular *D*_*v*_ = 0.1 Ns/m is in the range of values validated by Yoshikawa et al. (2016); *P*_*x*_ = 0.01 N/m satisfied the two requirements above, although its modification around that value was not critical.

## Results

The simulations of the modified intermittent control policy of the extreme-CIP model were labeled successful if the controller could prevent the stick from falling (|θ(*t*)| < π/4), while keeping the cart in the prescribed range (|*x*(*t*)| < 0.8 *m*), for a time interval of 2 min (plus an initial transient of 1 min). A given control model was supposed to generate at least 70% successful repetitions in order to be labeled stable.

The simulation experiments used the following set of parameters:

**Table:** 

Parameters for the simulation of the CIP model									
*L*	*M*	*m*	δ	*P*_θ_	*D*_ω_	*P*_*x*_	*D*_*v*_	σ_*noise*_	σ_*slope*_
Stick length [m]	Cart mass [kg]	Stick mass [kg]	Sensory delay [s]	Stick P control parameter [N/rad]	Stick D control parameter [Ns/rad]	Cart P control parameter [N/m]	Cart D control parameter [Ns/m]	Control additive noise [N]	Manifold Slope uncertainty
0.32	0.25	0.125	0.23	4–20	0.1686	0.01	0.1	0.015	0.2

**Figure 4:**
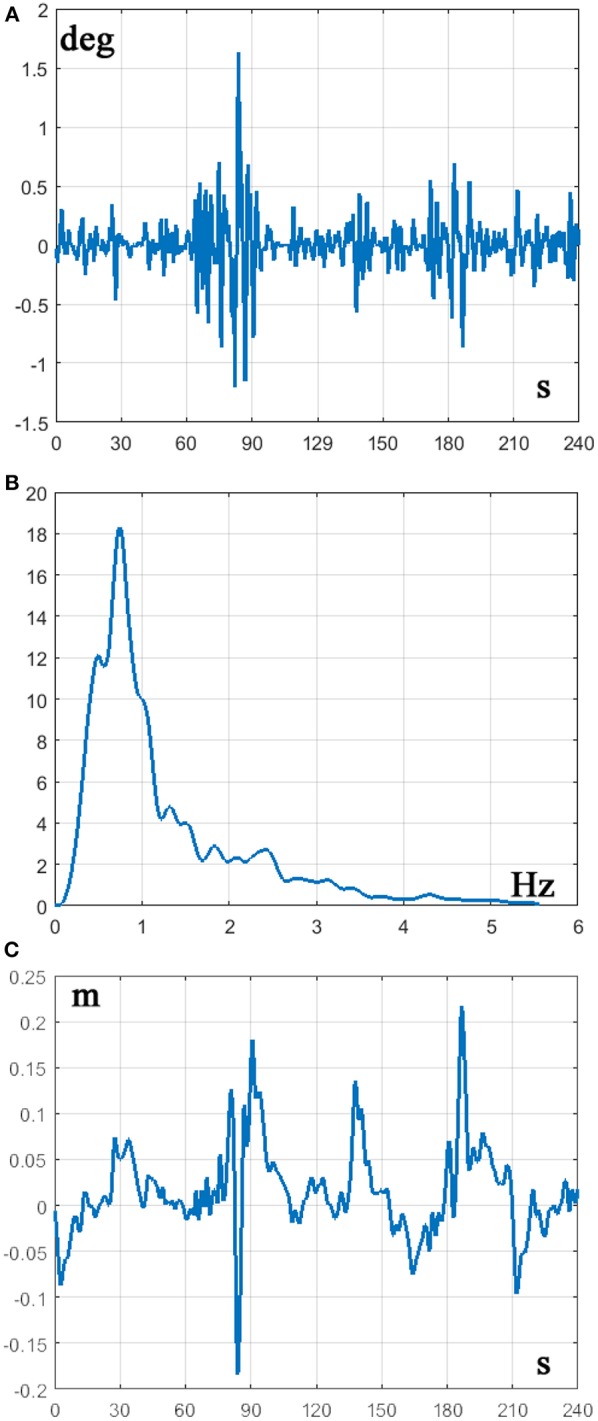
**(A)** Time series of the stick angle θ(*t*) during a 2 min balancing exercise. **(B)** Corresponding PSD. **(C)** time series of the cart displacement during the same time interval. CIP parameters: stick length *L* = 32 *cm*; cart mass *M* = 0.25 *kg*; Stick mass *m* = 0.125 *kg*; feedback delay δ = 230 *ms*. Controller parameters: *P*_θ_ = 5 *N*/*rad*; *D*_ω_ = 0.1826 *Ns*/*rad*; *P*_*x*_ = 0.01 *N*/*m*; *D*_*v*_ = 0.1 *Ns*/*m*.

**Figure 5:**
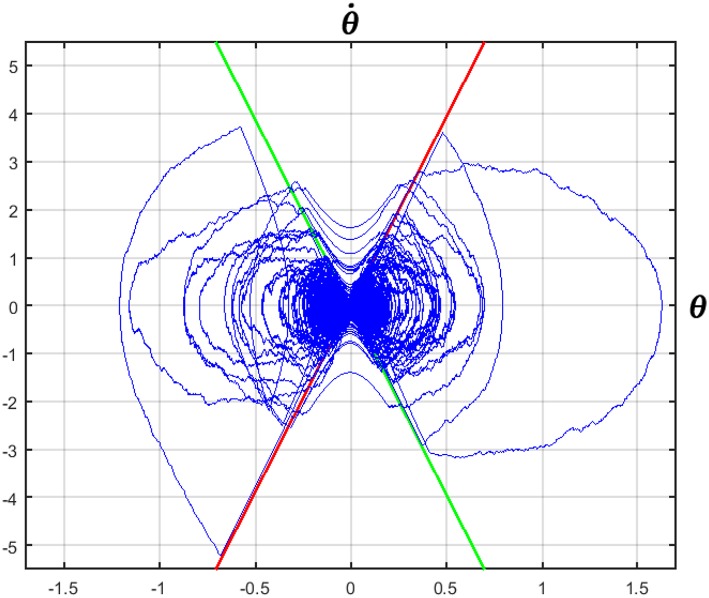
Phase portrait of a 2 min CIP balancing exercise with the modified intermittent control policy. CIP parameters: stick length *L* = 32 *cm*; cart mass *M* = 0.25 *kg*; stick mass *m* = 0.125 *kg*; feedback delay δ = 230 *ms*. Controller parameters: *P*_θ_ = 5 *N*/*rad*; *D*_ω_ = 0.1826 *Ns*/*rad*; *P*_*x*_ = 0.01 *N*/*m*; *D*_*v*_ = 0.1 *Ns*/*m*. The green and red lines correspond to the stable and unstable manifolds, respectively. Measurement units: deg vs. deg/s.

On the basis of the experience previously gained from the standard intermittent control policy of upright standing, we focused our attention on the *P*_θ_ control parameter in order to test the plausibility of the heuristic indication coming from Equation 22. We found indeed that if *P*_θ_ < 4 the intermittent controller failed in 100% of the simulation runs. However, a small of increase of *P*_θ_ was sufficient to stabilize the CIP in most of the cases. Figures 4, 5 show the result of a representative simulation performed with *P*_θ_ = 5.

**Figure 6:**
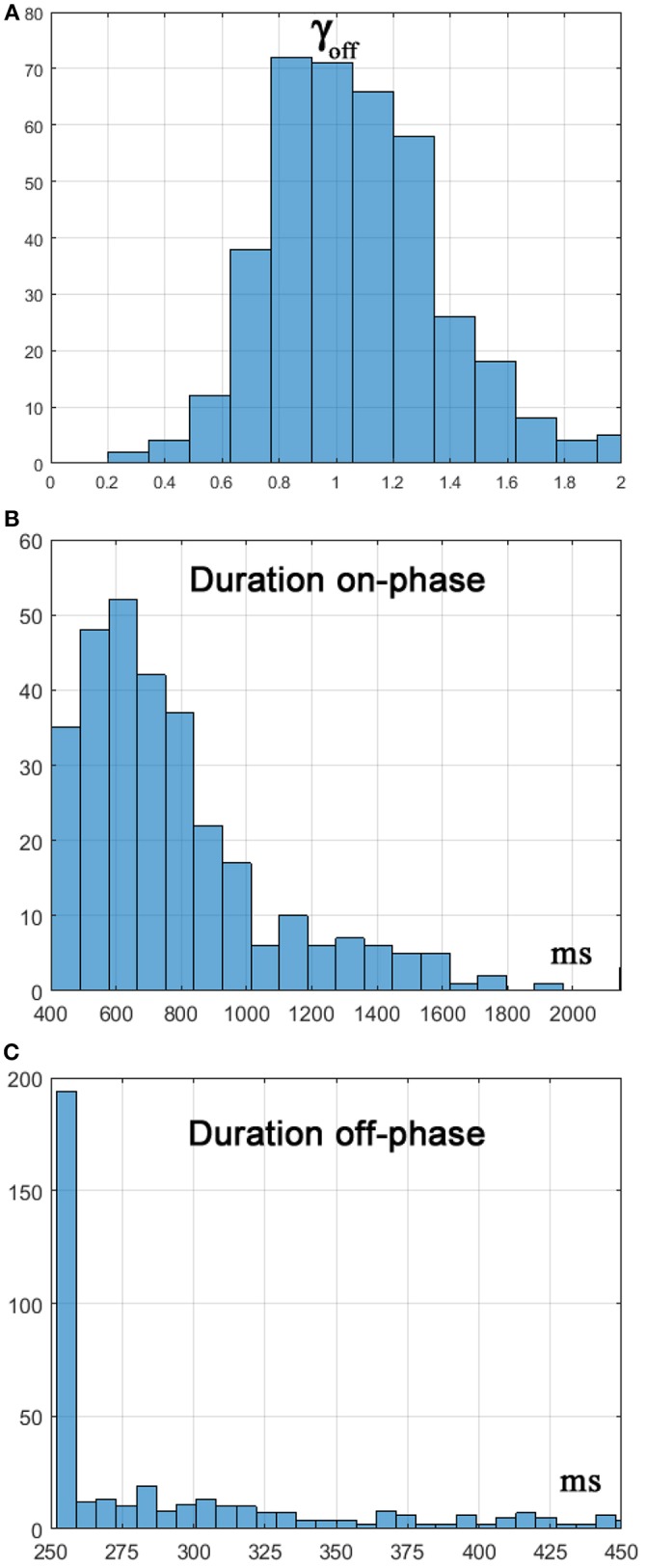
**(A)** Histogram of γ_*off*_, i.e., of the distance from the stable manifold of the state vector at *t* = *t*_*off*_. **(B)** Histogram of the duration of the on-phase. **(C)** Histogram of the duration of the off-phase.

Figure 4 displays the concurrent oscillations of the stick angle and the cart position, as well as the power spectral density (PSD) of the stick angle, characterized by a peak around 0.7 Hz, coherent with the experimental data of Yoshikawa et al. (2016) and (Milton et al., 2016). Figure 5 is a representative phase portrait of the stick oscillation, generated by the concatenation of hyperbolic off-phases and spiraling on-phases, disturbed more or less by the concurrent motion of the cart. Figure 6A shows the histogram of γ_*off*_ values that identify the distances of the state vector from the stable manifold, at the initial instant of each off-phase. The ideal value, in order to maximize the self-balancing action of the saddle-like instability, would be γ_*off*_ = 1; the histogram shows that the distribution of this indicator over a simulation trial is indeed centered around the target value. The other two panels of Figure 6 display the histogram of the duration of the on-phases and the corresponding histogram of the off-phases, respectively. The on-phases have generally a longer duration and are spread on a much larger range of values also as a consequence of the disturbing effect of the cart motion. In contrast, the off-phases are generally shorter and tend to cluster around a value a little bit higher than the sensory delay δ as a consequence of the phase-reset mechanism of the new intermittent control policy.

**Figure 7:**
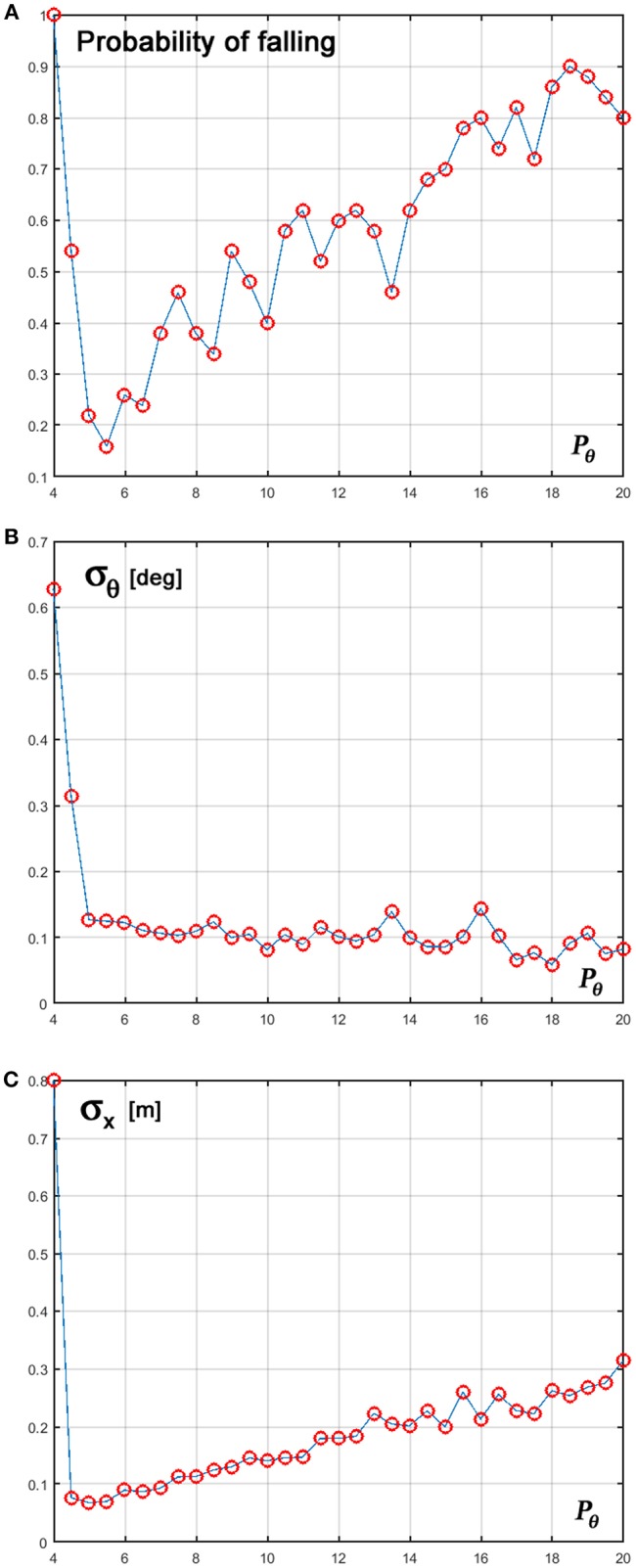
**(A)** Probability of falling over 100 repetitions; **(B)** Standard deviation of the stick oscillations; **(C)** Standard deviation of the cart motion, computed for the successful trials. The control parameter *P*_θ_ is varied between 4 and 20.

In order to evaluate the robustness of the new intermittent control policy we performed 100 simulations while changing the *P*_θ_ control parameter from 4 to 20. Sample tests were also performed for evaluating the sensitivity to variations of the other parameters without exhibiting any critical tuning problem. Figure 7 provides some evidence about the performance of the new control policy. Panel A shows that the probability of falling is 1 for *P*_θ_ < 4 but this value is quickly decreased to < 0.2 around a value of 5 where failure rate is minimal. For higher values of *P*_θ_ the failure rate progressively increases up to a value close to 100%. The other two panels show the standard deviation of the stick oscillations (panel B) and cart positions (panel C) averaged over the successful trials of the 100 repetitions. Remarkably, in spite of the increasing failure rate with greater values of *P*_θ_, the

range of the stick oscillation of the successful trials remains approximately stable. In contrast, there is a steady and significant increase of the amplitude of cart motion that is probably one of the reasons for the increasing failure rate. In summary, the reported experiments support the conclusion that the key control parameter should be tuned at the lowest possible value, just before the full-fledged establishment of uncontrolled instability.

We also evaluated the role of the uncertainty of the manifold slope, i.e., σ_*slope*_. With σ_*slope*_ = 0.2, namely a 20% uncertainly about the real value of the slope, the new control policy can indeed succeed to stabilize the CIP for the limit human case of a stick length of 32 cm. However, we also found that with such uncertainty level the control policy can indeed perform in a super-human manner, achieving successful stabilization for stick lengths as short as 26 cm. In order to clarify the point we performed simulations with σ_*slope*_ varying between 0.2 and 0.3 and found that al the highest uncertainty level (σ_*slope*_ = 0.3) the control policy fails in 100% of the simulations with a stick length of 32 cm. We also found that the human performance limit (90% success rate with a stick length of 32 cm) can be achieved with σ_*slope*_~0.25, i.e., with a 25% uncertainty of the slope of the unstable manifold. As previously remarked, this uncertainty incorporates also the inaccuracy of the internal simulation model as regards the selection of the termination time of the on-phase.

## Discussion

The simulation experiments performed in this study demonstrate that the basic rationale of the intermittent control policy, namely the exploitation of the intrinsic “affordance” of saddle-like dynamics during off-phases, is still plausible also for the extreme configuration of the CIP stabilization task, matching the human performance limit, with a modification that keeps the core computational outline based on an alternation of on-phases and off-phases. The additional computational process is a phase reset mechanism that provides a prediction capability, not in real-time and in a continuous manner (with a frequency band of the order of the kHz) but in specific time instants, at a rate of the order of 1 Hz.

In addition to the capability of matching the human performance limit in CIP balancing with a rather minor increase of the computational complexity of the standard intermittent control model, the new control policy is consistent with the experimental evidence (Milton et al., 2016) that the best performance in terms of successful CIP balancing trials is achieved by tuning the main control parameter near the edge of instability. Although this characteristic feature has been interpreted as evidence of a minimization of energetic costs, we doubt that the energetic issue is relevant in the specific case of CIP balancing with a very light apparatus like the one used by Yoshikawa et al. (2016) and the CIP model of this study. We evaluated indeed that the mechanical power required for balancing the model in the successful trials is quite small, of the order of 0.1 mW, on average, with brief power peaks, typically one or two per minute, never exceeding a fraction of a Watt. In alternative to such explanation, we suggest that tuning the proportional feedback parameter to the lowest possible value, before triggering uncontrolled unstable oscillations, is consistent with the general strategy of minimizing “stiffness” (in the most general sense) during the acquisition of a new skill, in the framework of a challenging learning process.

We need to stress that the new intermittent control model is not intended to substitute the standard version based on a threshold switching mechanism but should be considered as an extension made necessary for the human user when the challenge of the task is stretched to the limit of human performance. Without this motivation the simpler version of the control policy is the default choice: in that case, for the human user it is only necessary to tune a few control parameters and then freeze them during performance of the balancing task. We may speculate that when this strategy starts failing for the increased difficulty of the task the naïve user may attempt to extend it rather than substituting it with a completely different one. The logical key element that may attract the attention of the user is a more precise determination of the switching times, to be adapted at each oscillatory cycle, while inheriting all the dynamic features of the standard strategy that depend on the alternation of on-phases and off-phases. As already remarked, this additional computation, although somehow more complex than a simple threshold, has a limited bandwidth, related to the fine trimming of the sequence of transition times (*t*_*on*_, *t*_*off*_, *t*_*on*_, …), namely a few transitions per second. In particular, we suggested that this objective may be obtained by learning an internal model of the CIP dynamics paired with a phase-reset of the stick-state.

The limitations of the new control policy as well as the limitations of human performance are determined by the degree of uncertainty of the internal model components together with the noise of the feedback information about the state of the system. Ultimately, such sources of uncertainty are not important *per se* but for their effect on the inaccurate selection of *t*_*off*_: as a matter of fact, when the decision is taken to turn off the active control action, the state vector of the stick, whose real value has been approximated by the simulation of the internal model, may end up far away from its ideal target, namely the stable manifold of the CIP, whose slope is known with some uncertainty in any case. Therefore, what matters is not the precision *per se* of the state vector prediction generated by the simulation model or the accuracy *per se* of the estimate of the stable manifold slope but the overall inaccuracy of the relative position at *t*_*off*_ of the state vector with respect to the stable manifold, that we characterized with the γ_*off*_ indicator.

From the simulations we could also evaluate that the limits of human performance, namely the inability to balance a stick shorter than 32 cm, can be expressed as a 25% uncertainty about such relative position. A smaller level of uncertainty, say 20%, would allow a *super-human* performance limit, i.e., the ability to stabilize a CIP with a stick length as short as 26 cm; a higher level of uncertainty, say 30%, would a induce a degraded *sub-human* performance level. In any case, the acquisition of the relevant internal models (the geometric model of the stable manifold slope, the short-term sensorimotor memory for phase reset of the CIP state at *t*_*off*_, and the dynamic model for the on-phase simulation) imply a rather long learning process based on the acquisition and elaboration of a large number of unsuccessful trials: this well-reflects the fact that human subjects require indeed a large effort and long training, in order to become skilled performers at this level of challenge, whereas they almost immediately succeed to control the system in a less challenging situation, say a stick length of 1 m or more. Moreover, there are some subjects that persistently fail whatever the amount of training in the most challenging situation. Characterizing and modeling a learning process of this kind is clearly outside the purpose of this work, although we may investigate it in the near future: in any case, some suggestion may come from a preliminary study that focused on the use of reinforcement learning in relation with the emergence of intermittent-feedback control (Michimoto et al., 2016).

## Author Contributions

All authors listed have made a substantial, direct and intellectual contribution to the work, and approved it for publication.

### Conflict of Interest Statement

The authors declare that the research was conducted in the absence of any commercial or financial relationships that could be construed as a potential conflict of interest.

## Footnotes

^1^Although the Taylor series expansion of delayed terms in differential equations is not a well-defined mathematical procedure, it is a simple heuristic technique for obtaining order of magnitude evaluations whose plausibility can be checked by means of experiments or simulations.
