# Supplementary material for: Stabilization of a Cart Inverted Pendulum: Improving the Intermittent Feedback Strategy to Match the Limits of Human Performance
Source: Front Comput Neurosci. 2019 Apr 5;13:16. doi: 10.3389/fncom.2019.00016 (PMC6461063; doi:10.3389/fncom.2019.00016)
Supplement: Supplementary file 1 [file Data_Sheet_1.pdf]

Appendixes

Appendix 1: Derivation of the dynamics of the CIP system (eq. 1)

A simple way for deriving the CIP equations of motion is to follow the Lagrangian formulation for a 2 DoFs system with conservative driving forces:

$$\frac{d}{dt}\frac{\partial L}{\partial \dot{q}_i} - \frac{\partial L}{\partial q_i} = Q_i \qquad i = 1{,}2 \tag{A1}$$

characterized by two generalized coordinates $[q_1 = \theta, q_2 = x]$, the corresponding generalized forces $[Q_1 = 0, Q_2 = f(t)]$, and the Lagrangian function: $L(q, \dot{q}) = K(q, \dot{q}) - V(q)$.

By taking into account the parameters of the CIP model defined in figure 1, we can express the Lagrangian function in a straightforward manner:

$$K(q, \dot{q}) = \frac{1}{2}M\dot{x}^2 + \frac{1}{2}m(\dot{x}_{com}^2 + \dot{y}_{com}^2) + \frac{1}{2}I_{com}\dot{\theta}^2 \quad \text{with} \quad I_{com} = \frac{1}{12}mL^2$$

$$V(q) = mgy_{com} = mg\,\frac{L}{2}\cos\theta$$

$$\begin{cases} \dot{x}_{com} = \dot{x} + \frac{L}{2}\cos\theta\,\dot{\theta} \\ \dot{y}_{com} = -\frac{L}{2}\sin\theta\;\dot{\theta} \end{cases} \Rightarrow L(q, \dot{q}) = \frac{1}{2}(M + m)\dot{x}^2 + \frac{1}{6}mL^2\,\dot{\theta}^2 + \frac{1}{2}mL\cos\theta\,\dot{x}\,\dot{\theta} - mg\frac{L}{2}\cos\theta$$

Finally, by plugging this expression of the Lagrangian function into equation A1 we can derive immediately the equation of motion of the CIP, which is a second order non-linear ODE:

$$\begin{bmatrix}\ddot{\theta}\\ \ddot{x}\end{bmatrix} = \begin{bmatrix}A_{11}(\theta) & A_{12}(\theta)\\ A_{21}(\theta) & A_{22}(\theta)\end{bmatrix}\begin{bmatrix}\sin\vartheta\\ f\end{bmatrix}$$

$$\begin{cases} A_{11} = \dfrac{1.5}{L\left(M + m(1 - 0.75\ \cos^2\theta)\right)}\left((M+m)g - 0.5\,mL\,\dot{\theta}^2\cos\theta\right) \\ A_{12} = \dfrac{-1.5\ \cos\theta}{L\,(M + m(1 - 0.75\ \cos^2\theta))} \\ A_{21} = \dfrac{1}{M + m(1 - 0.75\ \cos^2\theta)}\left(0.5\,m\,L\,\dot{\theta}^2 - 0.75\,m\,g\cos\theta\right) \\ A_{22} = \dfrac{1}{M + m(1 - 0.75\ \cos^2\theta)} \end{cases} \tag{A2}$$

Appendix 2: Kinematics of the off-phase for the linearized model

When the delayed feedback control is switched off the linearized equation of the pendulum component of the CIP model is reduced to

$$\ddot{\theta} = A_{11}\,\theta \tag{A3}$$

Such model has two real eigenvalues of opposite sign $\lambda_{1,2} = \pm\sqrt{A_{11}}$ which characterize a saddle instability. The corresponding eigenvectors identify, in the phase plane ($\theta\ vs.\ \dot{\theta}$), an unstable manifold ($\dot{\theta} = \sqrt{A_{11}}\theta$), and a stable manifold ($\dot{\theta} = -\sqrt{A_{11}}\theta$). See figure A1.

In order to determine the trajectories generated by this model, starting from a generic initial position in the phase plane ($\theta_{off},\ \dot{\theta}_{off}$ at time $t = t_{off}$), we can consider the general solution of eq. A3:

$$\theta(t) = c_1 e^{\sqrt{A_{11}}\,(t-t_{off})} + c_2\,e^{-\sqrt{A_{11}}\,(t-t_{off})} \tag{A4}$$

and specialize it to the starting condition:

$$\begin{cases} c_1 = \dfrac{\dot{\theta}_{off} + \theta_{off}\sqrt{A_{11}}}{2\sqrt{A_{11}}} \\ c_2 = \dfrac{-\dot{\theta}_{off} + \theta_{off}\sqrt{A_{11}}}{2\sqrt{A_{11}}} \end{cases} \tag{A5}$$

We can then derive four families of trajectories in the phase plane of the system: two families of hyperbolic trajectories symmetric with respect to the horizontal axis and two families of hyperbolic trajectories symmetric with respect to the vertical axis

$$\dot{\theta} = \pm\sqrt{A_{11}(\theta^2 - {\theta_{off}}^2) + {\dot{\theta}_{off}}^2} \tag{A6}$$

These curves are separated by two asymptotic lines that correspond, respectively, to the unstable and stable manifolds defined above.

In general, if the initial position of the state vector is in the second or fourth quadrant of the phase plane, one part of each trajectory is converging to the saddle equilibrium point (the origin) and the other part is diverging, with a point at minimum distance where the trajectory intersects one of the two axes separated by a cross point, intersecting either the y-axis or the x-axis, at time t_c.

Computation of the time to cross.

Let us first define the following parameter that characterizes the starting point $[\theta_{off},\ \dot{\theta}_{off}]$ of a hyperbolic trajectory and is a measure of the distance of this point from the stable manifold ($\dot{\theta} = -\sqrt{A_{11}}\,\theta$):

$$\gamma_{off} = \left|\frac{\dot{\theta}_{off}}{\theta_{off}\sqrt{A_{11}}}\right| \tag{A7}$$

If $\gamma_{off} = 1$ the starting point is on the stable manifold, if $\gamma_{off} > 1$ it is outside, and if $\gamma_{off} < 1$ it is inside.

By considering eq. A4 and A5, for the trajectories symmetric with respect to the vertical axis ($\gamma_{off} > 1$) we can write $\theta(t_c) = c_1 e^{\sqrt{A_{11}}\ (t_c - t_{off})} + c_2\, e^{-\sqrt{A_{11}}\ (t_c - t_{off})} = 0 \ \Rightarrow \ c_1 e^{\sqrt{A_{11}}\ (t_c - t_{off})} = -c_2\, e^{-\sqrt{A_{11}}\ (t_c - t_{off})} \Rightarrow$ $e^{2\sqrt{A_{11}}\ (t_c - t_{off})} = -\frac{c_2}{c_1}$ and then we get the following explicit expression:

$$t_c - t_{off} = \Delta t_{cross} = \frac{1}{2\sqrt{A_{11}}} \ln\left(\frac{\gamma_{off} + 1}{\gamma_{off} - 1}\right) \tag{A8}$$

For the trajectories symmetric with respect to the horizontal axis ($\gamma_{off} < 1$) and we get a similar expression:

$$\Delta t_{cross} = \frac{1}{2\sqrt{A_{11}}} \ln\left(\frac{1+\gamma_{off}}{1-\gamma_{off}}\right) \tag{A9}$$

It is also possible to combine the two expressions of the crossing time in a single expression, whatever the value of γ_{off}:

$$\Delta t_{cross} = \frac{1}{2\sqrt{A_{11}}} \ln\left(\frac{1+\gamma_{off}}{|1-\gamma_{off}|}\right) \tag{A10}$$

This time strongly increases as the distance of the starting point from the stable manifold $|1-\gamma_{off}|$ decreases and diverges when it becomes zero. A crucial feature of this expression is that the time to cross does not depend on the initial angle per se but on the "distance" from the stable manifold, measured by the value of γ_0 (the distance is zero if γ_{off}=1). Figure 3 plots the time to cross as a function of γ_{off} for various values of the length L of the pendulum. The dotted line corresponds to a value of the time to cross of 230 ms.

Finally, by merging equations A4 and A5, it is possible to write the following formula in closed form

$$\begin{cases} \theta(t) = \dfrac{\dot{\theta}_{off} + \theta_{off}\sqrt{A_{11}}}{2\sqrt{A_{11}}}\, e^{\sqrt{A_{11}}\,(t-t_{off})} + \dfrac{-\dot{\theta}_{off} + \theta_{off}\sqrt{A_{11}}}{2\sqrt{A_{11}}}\, e^{-\sqrt{A_{11}}\,(t-t_{off})} \\ \dot{\theta}(t) = \dfrac{\dot{\theta}_{off} + \theta_{off}\sqrt{A_{11}}}{2}\, e^{\sqrt{A_{11}}\,(t-t_{off})} - \dfrac{-\dot{\theta}_{off} + \theta_{off}\sqrt{A_{11}}}{2}\, e^{-\sqrt{A_{11}}\,(t-t_{off})} \end{cases} \tag{A11}$$

which describes the full course of the stick trajectory in the off-phase and, in particular, allows to predict the state vector at the end of the off-phase, i.e. at $t = t_{on}$, as soon as the state vector at the beginning of the off-phase can be extracted from the short-term sensorimotor memory, i.e. at $t = t_{off} + \delta$.

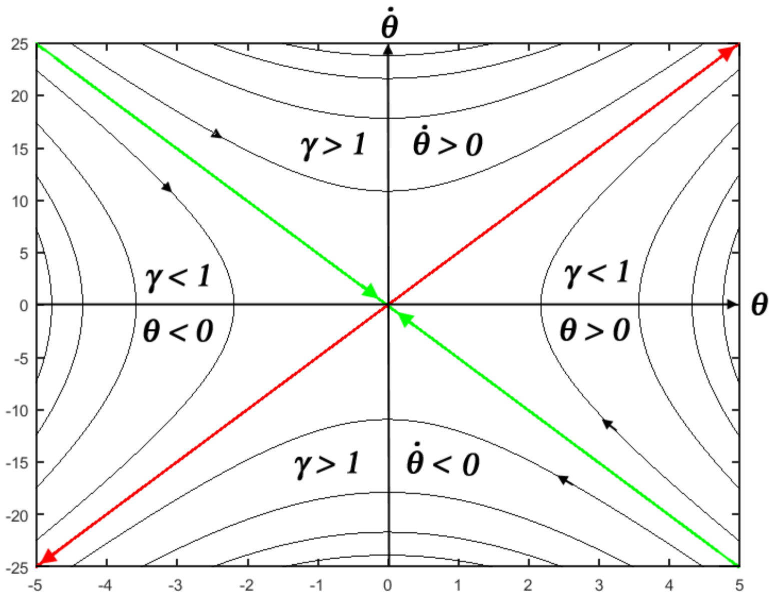

Figure A1. Phase plane characterization of the off-phase dynamics of the stick, highlighting the stable and unstable manifolds (colored green and red, respectively), and the four groups of hyperbolic trajectories: $(\gamma > 1, \dot{\theta} > 0)$, $(\gamma > 1, \dot{\theta} < 0)$, $(\gamma < 1, \theta > 0)$, $(\gamma < 1, \theta < 0)$.
